# The plasma concentration of VEGF, HE4 and CA125 as a new biomarkers panel in different stages and sub-types of epithelial ovarian tumors

**DOI:** 10.1186/1757-2215-6-45

**Published:** 2013-07-02

**Authors:** Sławomir Ławicki, Grażyna Ewa Będkowska, Ewa Gacuta-Szumarska, Maciej Szmitkowski

**Affiliations:** 1 Department of Biochemical Diagnostics, Medical University Białystok, Waszyngtona 15A, Białystok 15-269, Poland; 2 Department of Gynecology, Medical University, Białystok, Poland

**Keywords:** VEGF, HE4, CA125, Ovarian Cancer, Tumor Markers

## Abstract

**Background:**

VEGF may play a role in the pathogenesis of cancer disease, for example in cell growth, proliferation and angiogenesis. In this study, we investigated plasma levels of this cytokine in comparison to plasma levels of a new biomarker - HE4 and the established tumor marker CA125 in ovarian cancer patients (100) as compared to control groups: patients with a benign ovarian tumor (80) and healthy subjects (50).

**Methods:**

Plasma levels of VEGF were determined by ELISA, HE4 and CA125 by CMIA method.

**Results:**

The results showed that levels of VEGF, CA125 and HE4 were significantly higher in ovarian cancer (OC) patients as compared to the both control groups. VEGF has demonstrated as high as comparative markers values of the diagnostic sensitivity (SE), specificity (SP), the predictive values of positive and negative test results (PV-PR, PV-NR), and the area under the ROC curve (AUC) in early stages of cancer tested groups. The combined use of parameters studied resulted in the increase in the diagnostic criteria values and the AUC.

**Conclusions:**

These findings suggest the usefulness of VEGF in the early diagnostics of ovarian cancer, especially in combination with CA125 and HE4, as a new biomarkers panel. Additionally, VEGF is the most useful tool in the diagnostics of locally advanced ovarian cancer without metastases. Investigated cytokine presented similar to HE4 usefulness in differentiation of OC according to its histopathlogical sub-type, and could be used especially in the diagnostics of *endometrioid epithelial* OC.

## Background

Ovarian cancer (OC) is a highly lethal gynecological cancer. Approximately 23% of gynecologic cancers are ovarian in origin, but 47% of all deaths from cancer of the female genital tract occur in women with cancer of this organ. Malignant tumor of the ovaries occur at all ages with variation in histological sub-type by age [1,2]. Established risk factors for epithelial ovarian tumors include reproductive risk factors and inherited pathological mutations in the BRCA1 and BRCA2 genes [1,3,4]. OC lacks clear syndromes in the beginning, which prevents early discovery and treatment. Many potential biomarkers have been identified or used during last procedure for diagnostics of ovarian cancer patients [5,6]. Different types of proteins other than commonly accepted and used tumor marker - CA125 (carbohydrate antigen 125) [7-9] or a new biomarker - HE4 (human epididymis protein 4) [9,10] such as cytokines (M-CSF, IL-6) [11-14], and metalloproteinases are currently investigated [15-17].

Angiogenesis has been established as an important factor in human carcinogenesis influencing tumor growth and invasion [18]. Vascular endothelial growth factor (VEGF) which can be seen as the most important pro-angiogenic molecule, has been shown to parallel selective steps of tumor growth and the development of metastases [19,20] also through a direct, autocrine effect on tumor cells [21]. Breast and gynecologic cancer are among the best known malignancies that use lymphangiogenesis, the recruitment of blood and lymphatic vessels, to a growing tumor [22]. It was confirmed by other studies that VEGF plays an important role in the development of breast [23], reproductive organ [24-26] and ovarian cancer [27,28] as well as lung [29], colon [30], and gastric [31] cancer.

The aim of this study was to determine the plasma levels of vascular endothelial growth factor in comparison with the plasma levels of HE4 and CA125 in epithelial ovarian cancer patients in relation to the control groups: patients with a benign ovarian tumor and healthy subjects. Additionally, a comparison between plasma levels of the parameters tested with the stage of cancer, its histological sub-type and histological type of benign ovarian tumors were performed. Moreover, the diagnostic criteria (sensitivity, specificity, predictive values of positive and negative test results) and the receiver-operating characteristic curve (ROC) for the cytokine tested, HE4 and CA125 alone and in combinations were defined. Furthermore, a correlation between the parameters tested was established.

These data may be used in the evaluation of the usefulness of the VEGF in the diagnosis of stages and histological sub-types of ovarian cancer and in discrimination it from benign ovarian tumors, especially when analyzing with HE4 and CA125.

## Methods

### Patients

Table 1 shows the tested groups. The study included 100 epithelial ovarian cancer patients (sub-types *serous* and *endometrioid*) diagnosed by the Gynecology Group. Clinical stages and histological classification based on the criteria of the International Federation of Gynecology and Obstetrics (FIGO) and the World Health Organization (WHO) were established in all cases. The ovarian cancer histopathology was established in all cases by tissue biopsy of tumor or after surgery treatment from tumor cancer tissues. Patients with renal failure were excluded in our study because of very high HE4 concentrations levels, undistinguishable from ovarian cancer. None of the patients had received chemo- or radiotherapy before blood sample collection.

**Table 1:** Characteristics of ovarian cancer patients and control groups: benign ovarian tumor and healthy subjects

**Study group**		**Number of patients**
Epithelial ovarian cancer patients		100 (100%)
• median age (range)		59 (46–87)
- sub-type *serous epithelial*		54 (54%)
• median age (range)		59 (46–81)
- sub-type *endometrioid epithelial*		46 (46%)
• median age (range)		59 (49–87)
Tumor stage	IA-T_1a_N_0_M_0_	5 (5%)
	IB-T_1b_N_0_M_0_	7 (7%)
	IC-T_1c_N_0_M_0_	13 (13%)
	IIA-T_2a_N_0_M_0_	8 (8%)
	IIB-T_2b_N_0_M_0_	9 (9%)
	IIC-T_2c_N_0_M_0_	8 (8%)
	IIIA-T_3a_N_0_M_0_	9 (9%)
	IIIB-T_3b_N_0_M_0_	9 (9%)
	IIIC-T_3c_N_0_M_0_	7 (7%)
	IV(metastases)	25 (25%)
Menopausal status:		
**-** postmenopausal		100 (100%)
Benign ovarian tumor patients		80 (100%)
- type *cystis serous*		40 (50%)
- type *cystis endometriosis*		40 (50%)
Median age (range)		54 (48–68)
Menopausal status:		
- postmenopausal		80 (100%)
Healthy subjects		50 (100%)
Median age (range)		55 (47–64)
Menopausal status:		
- postmenopausal		50 (100%)

Pretreatment staging procedures included physical and blood examinations, ultrasound scanning and chest X-rays. In addition, radioisotope bone scans, the examination of bone marrow aspirates, and brain and CT scans were performed where necessary.

The control groups were comprised of 80 benign ovarian tumor patients (*cystis serous* or *cystis endometrioides*) and 50 healthy volunteers. The benign ovarian tumor histopathology was established in all cases by tissue biopsy of ovary tumor or after surgery.

The healthy women group was examined also by a gynecologist prior to blood collection and subjects with a clinical history of prior endometriosis or mild gynecological conditions were excluded. In addition, reproductive organ ultrasound scans or mammary ultrasound scanning were performed where necessary.

The ovarian cancer patients and the control group (benign lesions) were treated in the Department of Gynecology, University Hospital in Białystok, Poland, between 2006–2012 years. The study was approved by the local Ethics Committee of the Medical University in Białystok, numbers: R-I-002/314/2009 and R-I-002/262/2010 and all the patients gave their informed consent for the participation in the study.

### Biochemical analyses

Venous blood samples were collected from every patient. Blood was collected into a heparin sodium tube, centrifuged 1000 rpm for 15 min. to obtain plasma samples, and stored at –85°C until assayed. Tested cytokine (VEGF) was measured with the enzyme-linked immunosorbent assay (ELISA) (Quantikine Human HGFs Immunoassay, R&D systems), according to the manufacturer's protocols. Duplicate samples were assessed for each patient.

The intra-assay coefficient of variation (CV%) of VEGF is reported to be 4.5% at a mean concentration of 235 pg/ml, SD = 10.6. The inter-assay coefficient of variation (CV%) of VEGF to be 7.0% at a mean concentration of 250 pg/ml, SD = 17.4. The assay showed no significant cross-reactivity or interference with numerous human cytokines and other growth factors.

The plasma concentrations of CA125 and HE4 were measured by chemiluminescent microparticle immunoassay (CMIA) (Abbott, Chicago, IL, USA). The intra-assay CV for CA125 is reported to be 2.4% at a mean concentration of 43.5 U/ml, SD = 1.1. The inter-assay CV for CA125 - to be 3.9% at a mean concentration of 43.5 U/ml, SD = 1.7. The intra-assay CV for HE4 - to be 3.7% at a mean concentration of 39.0 pmol/L, SD = 1.4. The inter-assay CV for HE4 - to be 2.8% at a mean concentration of 39.0 pmol/L, SD = 1.1.

### Statistical analysis

The statistical analysis was performed using the STATISTICA 8.0 PL program. A preliminary statistical analysis (Chi-square test) revealed that the distribution of cytokine and tumor marker levels did not follow normal distribution. Consequently nonparametric methods were used to compare levels of tumor markers between the groups of patients. Comparisons between two groups were performed using the Mann–Whitney test. In case of multiple groups Kruskal-Wallis tests were calculated with post hoc comparisons according to Dwass-Steele-Critchlow-Fligner method. ROC analyses were utilized in the evaluation of diagnostic power of tumor markers. Markers were compared by assessing the significance of differences between the areas under their corresponding ROC curves. In addition, markers were compared by assessing the differences in sensitivity and specificity obtained for the optimal cut-off points. These points were determined using the criterion of maximizing the Youden coefficient. The construction of the ROC curves was performed using GraphRoc Program for Windows.

Data were presented as median and range. Statistically significant differences were defined as comparisons resulting in p < 0.05. The Spearman rank correlation was used in the correlation analyses.

The *cut-off* of VEGF (187.45 pg/ml), HE4 (75.75 pmol/L) and CA125 (28.89 U/ml) were calculated as 95^th^ percentile from the control group of healthy blood donors.

## Results

In the total group of ovarian cancer (OC) patients’ plasma levels of VEGF (168.48 pg/ml) and tumor markers, HE4 (103.60 pmol/L) or CA125 (133.30 U/ml) were found to be statistically higher compared to the healthy subjects (39.31 pg/ml; 44.15 pmol/L; 9.94 U/ml) (p < 0.001; respectively) (Table 2). Moreover we observed all the parameters’ concentrations significantly different comparing every stage of advancement (I-IV) of studied cancer with the same control group: I - p < 0.001 (VEGF and CA125), p = 0.0168 (HE4); II - p = 0.0187, p < 0.001, p = 0.0023; III - p < 0.001 (respectively); IV - p = 0.0281 (VEGF) p < 0.001 (CA125 and HE4). The plasma levels of mentioned above cytokine and tumor markers were also significantly different in the advanced stages (III-IV) than those found in the early stages (I-II): VEGF, CA125 in comparison of stage III with stage I (p = 0.0440; p < 0.001) and with II (p = 0.0430; p = 0.0028) or HE4, CA125 in comparison of stage IV with I (p < 0.001; p = 0.0032) and II (p = 0.0036; p = 0.0079) of tumor advancement. The concentrations of VEGF were statistically higher when comparing patients in stage III with stage IV (p = 0.0184). We have also noticed significantly higher (p = 0.0432) concentration of HE4 in OC patients sub-type *serous epithelial* compared to sub-type *endometrioid epithelial*.

**Table 2:** Plasma levels of VEGF, HE4 and CA125 in tested groups

**Groups**			**VEGF**	**HE4**	**CA125**
			**(pg/ml)**	**(pmol/L)**	**(U/ml)**
**Ovarian cancer Median Range**	**stage I**		^**1/2**^	^**1/2**^	^**1**^
			166.18	83.50	63.50
			15.38 -656.56	28.60-1093.80	12.00-557.20
	**stage II**		^**1/2**^	^**1/2**^	^**1/2**^
			170.50	62.60	61.45
			8.92-2701.07	27.00-625.10	9.80-998.00
	**stage III**		^**1/2/4**^	^**1/2/4**^	^**1/2/4**^
			345.50	117.90	766.60
			10.70-1429.50	48.70-1500.00	10.10-2060.78
	**stage IV**		^**1/2/4/5**^	^**1/2/4**^	^**1/2/4**^
			143.50	198.00	531.90
			9.00-1386.00	37.80-1944.20	14.30-8602.30
	**Total group**		^**1/2**^	^**1/2**^	^**1/2**^
			168.48	103.60	133.30
			8.92-2701.07	27.00-1944.20	9.80-8602.30
	***Serous epithelial***		^**1/6**^	^**1/6/7/9**^	^**1/6/7**^
			166.18	126.00	171.21
			8.92-1429.50	28.60-1944.20	9.80-8602.30
	***Endometrioid epithelial***		^**1/6**^	^**1/6/7**^	^**1/6/7**^
			184.24	68.45	114.00
			9.00-2701.07	27.00-1740.00	11.10-1425.00
**Control groups** Median Range	**Benign ovarian tumor*(Cystis)***	***Endometrioides***			^**3/8**^
			45.50	23.15	43.75
			9.00-560.50	14.00-65.60	7.50-2748.00
		***Serous***	^**3**^	^**8**^	^**3**^
			84.35	43.25	20.65
			9.00-2100.00	26.80-156.40	5.80-451.80
		**Total group**			^**3**^
			68.71	42.50	27.70
			9.00-2100.00	14.00-156.40	5.80-2748.00
	**Healthy subjects**		39.31	44.15	9.94
			2.30-467.10	6.20-122.30	5.06-36.60

^**1**^statistically significant when comparing OC patients with healthy subject.

^**2**^statistically significant when comparing OC patients with benign ovarian tumor total group.

^**3**^statistically significant when comparing patients with benign ovarian tumor and healthy subject.

^**4**^ statistically significant when comparing OC patients in stage III or IV with stage I or stage II.

^**5**^ statistically significant when comparing OC patients in stage IV with stage III.

^**6**^ statistically significant when comparing with benign ovarian tumor group. i.e. type *cystis endometrioides*.

^7^statistically significant when comparing with benign ovarian tumor group. i.e. type *cystis serous*.

^8^ statistically significant when comparing patients with benign ovarian tumor. i.e. type *cystis endometrioides* with type *cystis serous*.

^9^ statistically significant when comparing OC patients. i.e. sub-type *serous epithelial* with sub-type *endometrioid epithelial*.

Furthermore, we have observed statistical differences of the results for VEGF, HE4 and CA125 between groups with *serous epithelial* (166.18 pg/ml; 126.00 pmol/L and 171.21 U/ml) or *endometrioid epithelial* sub-types of OC (184.24 pg/ml; 68.45 pmol/L and 114.00 U/ml) and with healthy control (Table 2).

Patients with ovarian cancer (total group) had statistically considerably higher levels of researched factors (VEGF p = 0.0011; HE4 p < 0.001; CA125 p < 0.001) than those observed in benign ovarian tumor patients (Table 2). We noticed also similarly significantly higher concentrations of VEGF in I-IV (p = 0.044; p = 0.045; p = 0.0001 and p = 0.044; respectively), of HE4 in I-IV stages (p = 0.0184; p = 0.0014; p < 0.001 and p < 0.001), and of CA125 in II-IV (p = 0.0341; p < 0.001 and p < 0.001) stages of OC than in total benign lesion group. The following observation of these two compared groups revealed that the distribution of all tested factors among two histological sub-types of cancer and among non-carcinoma lesions patients (*cystis*) were significantly different.

Additionally the median levels of CA125 in the two histopathological sub-groups as well as in the total group of benign lesions (*cystis*) were statistically higher than in healthy subjects group (p < 0.001 in all cases). VEGF obtained similar results but only in *cystis serous* group (p = 0.0493). We have noticed also significant differences in the concentrations of tumor markers (HE4; CA125) when compared *cystis endometrioidess* (23.15 pmol/L; 43.75 U/ml; respectively) to *cystis serous* group (43.25 pmol/L; 20.65 U/ml; respectively).

Table 3 presents the diagnostic criteria of parameters tested: sensitivity (SE), specificity (SP), predictive value of a positive (PV-PR) and negative (PV-NR) test result in the OC patients. We observed higher ranges of the SE of VEGF and tumor markers in more advanced ovarian tumor stages (exception – HE4 in II stage and VEGF in IV stage). They were the highest for CA125. However, VEGF and HE4 had better results in the group with stage I and comparable results in the group with stage III (with stage IV only for HE4) (Table 3). The combined use of VEGF and tumor markers resulted in an increase in the diagnostic SE to very high range in stage I: 76% and 72% for the combination of VEGF with tumor markers (HE4, CA125), up to 84% (for VEGF + HE4 + CA125). The maximum ranges (96%) were obtained for the combinations of all parameters tested in stages II-IV and in the total OC group (93%) (Table 3). We have noticed also the highest ranges of diagnostic sensitivity according to histological sub-type of OC: the *serous epithelial* group for HE4 (70%), for the combination of HE4 and VEGF (81%) and of all parameters (94%) and in the *endometrioid epithelial* group for CA125 (69%), for the combination of CA125 and VEGF (91%), and of all parameters as well (95%).

**Table 3:** The diagnostic criteria of VEGF tested and in combination analysis with HE4 and CA125 in ovarian cancer patients

**Epithelial ovarian cancer**	**Diagnostic criteria (%)**	**VEGF**	**HE4**	**CA125**	**VEGF + HE4**	**VEGF + CA125**	**HE4+ CA125**	**VEGF + HE4 + CA125**
**I stage**	Sensitivity	44	44	40	76	72	72	84
	Specificity	94	94	92	88	88	88	82
	PV-PR	78	78	71	76	75	75	70
	PV-NR	77	77	75	88	86	86	91
**II stage**	Sensitivity	48	28	64	64	76	80	96
	Specificity	94	94	92	88	88	88	82
	PV-PR	75	70	80	72	76	76	72
	PV-NR	74	72	83	83	88	89	97
**III stage**	Sensitivity	72	68	80	88	92	88	96
	Specificity	94	94	92	88	88	88	82
	PV-PR	85	85	83	78	79	78	72
	PV-NR	87	85	90	93	95	93	97
**IV stage**	Sensitivity	40	80	84	88	92	92	96
	Specificity	94	94	92	88	88	88	82
	PV-PR	76	86	84	78	79	95	72
	PV-NR	75	85	92	93	95	79	97
**Total group**	Sensitivity	48	55	67	81	83	83	93
	Specificity	94	94	92	88	88	88	82
	PV-PR	94	94	94	93	93	93	91
	PV-NR	47	51	58	69	72	72	85
***Serous epithelial***	Sensitivity	46	70	64	81	77	88	94
	Specificity	94	94	92	88	88	88	82
	PV-PR	89	88	92	88	87	88	95
	PV-NR	71	74	71	81	78	88	93
***Endometrioid epithelial***	Sensitivity	50	36	69	71	91	80	95
	Specificity	94	94	92	88	88	88	82
	PV-PR	88	80	91	84	87	86	83
	PV-NR	67	61	77	77	93	83	95

The cytokine tested and tumor markers diagnostic specificities (SP) presented high values: 94% - VEGF and HE4; 92% - CA125. The combined use of studied factors resulted in a decrease in the diagnostic SP (Table 3).

All parameters tested showed high predictive values of a positive test result – PV-PR and a negative test result – PV-NR in all the groups investigated. The PV-PR in the total group of OC patients had very high and equal values (94%) for all the parameters tested, the PV-NR was the highest for CA125 (58%). The combined use of VEGF and HE4 with CA125 for the remaining group resulted in a decrease in the PV-PR (91%) and in an increase in the PV-NR (85%) values (Table 3). However, a maximum range of PV-PR (as high as 95%) was obtained for the combination of two tumor markers in stage IV and for the combination of three parameters investigated in *serous epithelial* ovarian cancer group. Similarly, a maximum range of PV-NR (97%) was obtained also for the combination of all factors in stages II, III and IV of ovarian cancer.

The Spearman’s rank correlation was used in the analyzes of dependence between the investigated parameters (data not shown, Additional file 1). There were significant correlations in the OC group: negative between HE4 and CA125 concentrations (R = −0.41; p = 0.036) in II stage and positive between VEGF and HE4 (R = 0.28; p < 0.001), VEGF and CA125 (R = 0.38; p < 0.001) and also between HE4 and CA125 concentrations (R = 0.32; p = 0.001) in the total group. Statistically significant positive correlations were also observed between the HE4 and CA125 levels in the *serous epithelial* (R = 0.35; p = 0.008) and *endometrioid epithelial* (R = 0.31; p = 0.037) ovarian cancer patients. Furthermore, a significant positive correlations were noticed between the VEGF and CA125 concentrations in the benign ovarian lesions group: *cystis serous* as well as in total group (R = 0.39; p = 0.012 and R = 0.23; p = 0.040; respectively). Similarly, significant positive correlation between the VEGF and HE4 levels in the healthy control group (R = 0.35; p = 0.012) was showed.

The relationship between the diagnostic SE and SP was illustrated by the ROC (receiver-operating characteristics) curve. Table 4 shows the results of our in-depth analysis of the AUC (area under ROC curve) of every parameter alone and in combination for every stage and for every investigated histological sub-type of the disease. It shows that the CA125 area (0.9276) under the ROC curve is the largest in the total group and both in the groups with *serous epithelial* (0.9047) and *endometrioid epithelial* (0.9128) sub-type (Table 4). The AUC of CA125 is the largest also in the group of patients with stage II (0.8652) and IV (0.9472), but interestingly the AUC of VEGF is the largest in the group with stage I (0.7984) and of HE4 with stage III tumor (0.9256). In addition, the AUC of cytokine tested and HE-4 presented distinctly the increase with stage of advancement of OC (with exception of VEGF in IV stage), identically as CA125. The combination of all parameters resulted in a further increase in the area under the ROC curve in every case, to the value: 0.8990 in I stage; 0.9207 in II stage; 0.9600 in IV stage, 0.9404 in total group, the maximum value was obtained in III stage (0.9837). Similarly, the areas under the ROC curve were also the largest for the combination of three parameters in each cancer sub-type group (0.9395; 0.9414). Moreover, the AUCs of the VEGF or commonly accepted tumor markers were significantly higher compared to AUC = 0.5 in every group studied of OC (Table 4).

**Table 4:** The diagnostic criteria of the ROC curve for VEGF in combination with HE4 and CA125 in ovarian cancer patients

**Epithelial ovarian cancer**	**The ROC criteria**	**VEGF**	**HE4**	**CA125**	**VEGF + HE4**	**VEGF + CA 125**	**HE4 + CA125**	**VEGF + HE4+ CA125**
**I stage**	AUC	0.7984*	0.7284*	0.7652*	0.8632*	0.8784*	0.8327*	0.8980*
	SE	0.0562	0.0708	0.0416	0.0508	0.0475	0.0538	0.0433
	95% C.I.	0.688-0.909	0.590-0.867	0.691-0.794	0.764-0.963	0.785-0.971	0.727-0.938	0.813-0.83
	*p* AUC = 0.5	**<0.001**	**0.0013**	**0.0005**	**<0.001**	**<0.001**	**<0.001**	**<0.001**
**II stage**	AUC	0.7231*	0.7646*	0.8652*	0.8592*	0.8587*	0.9207*	0.9207*
	SE	0.0647	0.0562	0.0389	0.0421	0.0510	0.0314	0.0294
	95% C.I.	0.596-0.850	0.654-0.875	0.814-0.967	0.777-0.942	0.759-0.959	0.859-0.982	0.863-0.978
	*p* AUC = 0.5	**0.0006**	**<0.001**	**<0.001**	**<0.001**	**<0.001**	**<0.001**	**<0.001**
**III stage**	AUC	0.9012*	0.9256*	0.9112*	0.9680*	0.9665*	0.9763*	0.9837*
	SE	0.0428	0.0291	0.0428	0.0167	0.0221	0.0302	0.0120
	95% C.I.	0.817-0.985	0.869-0.983	0.827-0.995	0.935-1.001	0.923-1.010	0.902-1.020	0.960-1.007
	*p* AUC = 0.5	**<0.001**	**<0.001**	**<0.001**	**<0.001**	**<0.001**	**<0.001**	**<0.001**
**IV stage**	AUC	0.7168*	0.9124*	0.9472*	0.9240*	0.9494*	0.9420*	0.9600*
	SE	0.0722	0.0428	0.0260	0.0382	0.0315	0.0353	0.0281
	95% C.I.	0.575-0.858	0.829-0.996	0.896-0.998	0.849-0.999	0.888-1.011	0.873-1.011	0.905-1.015
	*p* AUC = 0.5	**0.0027**	**<0.001**	**<0.001**	**<0.001**	**<0.001**	**<0.001**	**<0.001**
**Total group**	AUC	0.7843*	0.8321*	0.9276*	0.9032*	0.9127*	0.9180*	0.9404*
	SE	0.0376	0.0321	0.0221	0.0239	0.0250	0.0223	0.0187
	95% C.I.	0.711-0.858	0.769-0.895	0.884-0.971	0.856-0.950	0.864-0.962	0.874-0.962	0.904-0.977
	*p* AUC = 0.5	**<0.001**	**<0.001**	**<0.001**	**<0.001**	**<0.001**	**<0.001**	**<0.001**
***Serous epithelial***	AUC	0.7522*	0.8720*	0.9047*	0.9124*	0.9009*	0.9391*	0.9395*
	SE	0.0482	0.0347	0.0301	0.0278	0.0312	0.0233	0.0230
	95% C.I.	0.658-0.847	0.804-0.964	0.846-0.964	0.858-0.967	0.840-0.962	0.893-0.985	0.894-0.985
	*p* AUC = 0.5	**<0.001**	**<0.001**	**<0.001**	**<0.001**	**<0.001**	**<0.001**	**<0.001**
***Endome-trioid epithelial***	AUC	0.8226*	0.7843*	0.9128*	0.8922*	0.9268*	0.8926*	0.9414*
	SE	0.0438	0.0467	0.0305	0.0323	0.0299	0.0330	0.0228
	95% C.I.	0.737-0.908	0.693-0.876	0.853-0.973	0.829-0.955	0.868-0.985	0.828-0.957	0.897-0.986
	*p* AUC = 0.5	**<0.001**	**<0.001**	**<0.001**	**<0.001**	**<0.001**	**<0.001**	**<0.001**

C*.I.* confidence intervals of AUC.

* statistically significant when comparing tested parameters AUC’s with 0.5 AUC’s.

## Discussion

Establishment of an appropriate screening test for ovarian cancer has long been sought. Symptoms that are associated with this malignancy are typically nonspecific and the association is often not recognized until the disease is advanced [32]. Elevated serum CA125 levels have been detected in 50% and 92% of OC in early and late stages, respectively [33], similarly to the results observed for HE4 [34]. Several studies have aimed to explore the link between serum VEGF levels and ovarian cancer [27,28].

In this study, we investigated the diagnostics usefulness of VEGF and in combination with HE4 and CA125 in the patients with epithelial ovarian malignancies. Analysis of the usefulness of cytokine alone or in combination with other tumor markers, may improve the effectiveness of non-invasive diagnostics of this cancer [35]. Furthermore, we made a comparison of the received results with the results of the tumor markers and with the control groups (benign ovarian lesions patients and healthy subjects). Additionally, we estimated the diagnostic utility of mentioned above parameters in correlation to the stage of ovarian cancer.

Our results show that VEGF concentrations as well as HE4 and CA125 levels in all groups of ovarian cancer patients were statistically significantly higher in comparison to the group of healthy subjects and patients with benign tumors (exception CA125 I stage). These data are very similar to the studies of other authors, who compared patients with ovarian [36-40] or breast cancer [41] with healthy volunteers however, there were differences in the number of patients. Our results are consistent with the results of Sedlakova et al. [40] and Li et al. [39] who observed increased levels of VEGF in the group of ovarian cancer patients compared to the benign ovarian tumor patients, although the tested groups with non-malignant lesions were much smaller (15 and 25 women respectively). Similar results were also received by Cheng and others [42] who observed higher concentrations but of VEGF-C in ovarian cancer (109 patients) in comparison to different benign gynecological lesions (76 subjects). In opposition to our observations there was no difference in the VEGF concentrations between OC patients and benign tumors group although only 7 serous malignant and 3 borderline tumors were included [43]. Significantly higher serum levels of CA125 and HE4: (p = 0.005) [44] or (p < 0.0001) [38,45] were found in OC patients than in those with benign diseases though there was various composition and menopausal status of the groups compared.

Moreover, it was observed that VEGF concentrations, similarly to HE4 and CA125, were statistically higher in every group (the analysis related to the stage of advancement of ovarian cancer), compared to the healthy controls. Similar results were confirmed by different authors [42], though they observed the highest concentrations of VEGF-C in more advanced stages and various sub-types of OC were enrolled in the study (*serous-papillary*, *mucinous*, *endometrioid*, *clear cell*). Interestingly, it was found that VEGF concentrations in serum significantly surpassed the control level in breast cancer patients (stages I-II) [23]. Others found circulating concentrations of HE4 and CA125 significantly higher in patients with stage IA-IIB compared with healthy women, though more than four sub-types of ovarian cancer were included in the investigation and there was different the ethnical characteristics of the population selected [36].

Furthermore, significantly elevated levels of all parameters tested in the groups with early and late stage of ovarian cancer were observed compared to the cyst group. Our results are in agreement with different publications about our studied comparative tumor markers [36,46,47]. We were unable to confirm findings about VEGF in the papers published, since no reports on the subject are available.

The analysis of research results from literature revealed also almost identical to our observations significant differences when comparing patients in more advanced stage to metastasis-free early stage [36,39,40,46], and is in opposition to another publication where a half smaller group of postmenopausal women was studied [48]. It should be underlined also that in our investigations VEGF showed a significant difference in the concentrations between the two groups with more advanced stage of OC (III and IV – higher values for III stage). This may indicate the highest diagnostic usefulness of VEGF in locally advanced and metastases free stages of OC. One possible explanation for relatively low levels in patients with IV stage could be that VEGF is rapidly metabolized and excreted in case of presence many small, metastatic tumors. To our knowledge, little is known about the pharmacokinetic or pharmacodynamic characteristics of this cytokine. Undoubtedly, VEGF plays an essential role in the growth and metastases of ovarian cancer. Mattern and colleagues [49] showed the close correlation of VEFG expression with tumor cell proliferation. Vascular endothelial growth factor may also contribute to ovarian cancer metastasis by directly stimulating proliferation, survival, and/or migration of tumor cells [50]. It has been validated by others that serum VEGF was an independent prognostic parameter in a large series of patients with all stages of OC [27].

To date no large studies had examined VEGF levels in healthy postmenopausal women with benign gynecological disorders. Cheng and colleagues [42] found no differences between benign ovarian diseases and healthy controls for serum levels of VEGF-C and CA125 (p > 0.05), though patients with *cystis serous* or *cystis endometrioides* were not enrolled in the group studied.

In opposition, we found in our previous study significant higher concentrations of CA125 in the group of 70 postmenopausal women with benign lesions of the ovary (cysts) [51]. Our present observations about both tumor markers are in agreement with published evidence [47].

We showed the VEGF concentrations significantly different in the patients with studied sub-types of OC vs patients with *cystis endometrioides*, though we did not observe statistical differences in the concentrations of mentioned above cytokine between the two control groups: patients with *cystis endometrioides* and the healthy women group. On the basis of obtained results the VEGF could be presented as a useful diagnostic tool for the differentiation of patients with OC sub-type *endometrioid epithelial*, though we can not confirm these findings by other publications. Similarly to the discussed above results, HE4 also presented usefulness in the diagnostics of both investigated sub-types of OC. Moreover, patients with *serous epithelial* OC had significantly higher plasma levels of HE4 than patients with *endometrioid epithelial* OC. Patients with benign *endometrioid epithelial* ovarian tumors had lower HE4 and higher CA125 concentrations than patients with benign *serous epithelial* ovarian tumors. These data are in variance with previous observations [44,52], and may be in part affected by the patients’ selection, though other studies have recently reported similar results [46]. In agreement with the publications of Li et al. [39] and Czekierdowski et al. [48] we found no significant differences in VEGF levels among different pathological sub-types of ovarian carcinoma.

The correlation between examined markers levels was estimated by Spearman’s rank correlation test, because the value of each marker was not in a parametric distribution. Most of the measured values tended to be increased for every marker and in opposition to the publications of Tan and colleagues [53] we found a positive correlation between preoperative VEGF and CA125 levels, similarly to the other publication (r = 0.44; p = 0.0015) [43], though Manher et al. [54] found these parameters inversely correlated (r = −0.460; p = 0.01). Furthermore, we found positive correlation between these two mentioned above parameters also in control group (cysts) in opposition to the results of others (r = 0.10; p = 0.28) [43] and between VEGF and HE4 in healthy individuals group but no publications could confirm our findings. In accordance to demonstrated in our study results some authors showed positive correlation between CA125 and HE4 levels among patients with ovarian carcinoma (r = 0.54 p < 0.0001) [37,55].

The ability to detect early cancers would definitely improve patient prognosis. It was demonstrated that the diagnostic sensitivity increased with the progress of cancer disease and was the highest for CA125 (from 40% in stage I up to 84% in stage IV of the disease). Our results are in agreement with the literature [33]. On the other side, we could not demonstrate a better sensitivity than CA125 for ovarian cancer by alone VEGF testing, neither in early stages nor in advanced stages (though SE of this cytokine reached equal or even higher values to HE4 in particular stages). This could be explained by other authors’ investigations that levels of detectable VEGF in serum may depend on the binding to vascular endothelium or soluble receptors and/or degradation. In addition, VEGF levels in serum may also depend on the biosynthesis and release of VEGF from blood mononuclear cells and activated platelets [56]. It is interesting to point out that we found out a maximum increase in the diagnostic sensitivity for the combination of all parameters tested to 84% in stage I, even to 96% in stages II-IV, and to 94-95% in every histological sub-type of OC. Results of this study suggest that combining VEGF with CA125 and HE4 measurements might enable improved early detection of this cancer as compared with use of either marker alone or of both comparative tumor markers. This observation is in accordance to our previous papers, in which we evaluated diagnostic criteria of selected cytokines and commonly used tumor markers in various gynecological malignancies [51,57,58]. Several studies have found sensitivity to be greater than in either marker alone: VEGF and CEA (carcinoembrionic antigen) in colorectal [59] or HE4 and CA125 in ovarian cancer patients [5,45]. Diagnostic specificity (SP) was very high and reached the value of 94% for VEGF and HE4, and 92% for CA125. We found similar results in the existing literature [37,39,44].

The positive predictive value of the parameters tested were high in the total group of OC (94%). The negative predictive value shows the ability to exclude malignant disease and was the highest for CA125 (58%) in the total group of OC, though in current study the combination of both comparative tumor markers or with VEGF had unquestionable higher value (95%). These observations are in agreement with those recently reported by Molina et al. [44] and in opposition to the results of other authors who observed higher values of PV-NR for CA 125 or HE4 alone (92% and 72%; respectively) in Asian women [60], or distinctly higher values of these diagnostic criteria for HE4 than CA125 though for comparison of epithelial OC vs various benign lesions of the ovary in premenopausal women [61]. We were unable to compare our findings about VEGF by the papers published, since no reports on the subject are available. The results of the current study support our previous findings regarding another cytokine - M-CSF in this malignancy [51].

The area under the ROC curve (AUC) indicates the clinical usefulness of a tumor marker. Our results show that CA125 had the highest diagnostic power from the parameters tested in the total group of OC patients (AUC = 0.9276) and its value was negligibly higher than that of HE4 (AUC = 0.8321). Interestingly, VEGF had the largest AUC (0.7984) in the patients with I stage, and comparable to HE4 in patients with II and III stage. Diagnostic accuracy was increased when three parameters were included resulting in the highest diagnostic power in every group studied regarding to the stage and histological sub-type of epithelial OC. Furthermore, we observed that the areas under the curve for VEGF and comparative markers were statistically significantly larger compared to AUC = 0,5 - borderline of diagnostic usefulness of the test. Test data published by Candido dos Reis and colleagues [43] indicated area under the ROC curve – 0.99 for VEGF in diagnosis of epithelial ovarian tumors, though the cyst fluid was used as a material for the assay. In contrary to the current results Cheng et al. [42] found the AUC of VEGF-C larger than CA125 in different screening groups: OC versus benign ovarian diseases and healthy controls (0.826; 0.760; respectively), OC vs healthy individuals (0.862; 0.853) and finally OC vs benign ovarian diseases (0.802; 0.681). In a few previous studies the AUC values of CA125 and HE4 for differentiating ovarian cancer were 0.82-0.95 and 0.85-0.96, respectively [37,38,45-47,62] and was significantly higher for the combination of mentioned above markers [62] which were similar to our findings. Slight differences of the results between the studies might be caused by differences in the number of patients and the histological types or disease stages of ovarian cancer enrolled in each study.

## Conclusions

In summary, to the authors’ knowledge, the diagnostic usefulness of VEGF independently and especially in combination with both established ovarian tumor markers has not been previously described in the literature. Results of this study suggest that combining VEGF, HE4 and CA125 measurements might enable improved early detection of OC as compared with use of either marker alone. Additionally, VEGF is the most useful tool in the diagnostics of locally advanced ovarian cancer without metastases. Investigated cytokine presented similar to HE4 usefulness in differentiation of OC according to its histopathlogical sub-type, and could be used especially in the diagnostics of *endometrioid epithelial* OC.

## Abbreviations

AUC: Area under ROC curve; CA 125: Carbohydrate antigen 125; CEA: Carcinoembrionic antygen; CMIA: Chemiluminescent microparticle immunoassay; CT: Computed tomography; ELISA: Enzyme-linked immunosorbent assay; FIGO: International Federation of Gynecology and Obstetrics; HE4: Human epididymis protein 4; HGFs: Hematopoietic growth factors; IL-6: Interleukin 6; M-CSF: Macrophage-colony stimulating factor; OC: Ovarian cancer; PV-PR: Predictive value of a positive test result; PV-NR: Predictive value of a negative test result; ROC: Receiver-operating characteristics; SE: Diagnostic sensitivity; SP: Diagnostic specificity; TNM: Tumor-nodulus-metastasis classification; VEGF: Vascular endothelila growth factor; WHO: World Health Organization.

## Competing interests

The authors declare that they have no competing interests.

## Authors’ contributions

SŁ conceived of the study, carried out the immunoassays, performed the statistical analysis and drafted the manuscript. GEB carried out the immunoassays and helped to draft the manuscript. EGS carried out the acquisition of data and participated in the sequence alignment. MS participated in the design and coordination of the study and interpretation of data. All authors read and approved the final manuscript.

## Authors’ information

This study is a continuation of our research programme concerning cancer diseases of the breast and the reproductive organs, of which several previous manuscripts have been published in journals last years.

## Supplementary Material

Additional file 1: The Spearman’s rank correlation. Click here for file
